# Rabson-Mendenhall Syndrome: Analysis of the Clinical Characteristics and Gene Mutations in 42 Patients

**DOI:** 10.1210/jendso/bvae123

**Published:** 2024-06-16

**Authors:** Wenfeng Gong, Wenzhe Chen, Jianjun Dong, Lin Liao

**Affiliations:** Department of Endocrinology and Metabology, The First Affiliated Hospital of Shandong First Medical University and Shandong Provincial Qianfoshan Hospital, Shandong First Medical University, Shandong Key Laboratory of Rheumatic Disease and Translational Medicine, Shandong Institute of Nephrology, Jinan, 250014, China; Department of Endocrinology and Metabology, The First Affiliated Hospital of Shandong First Medical University and Shandong Provincial Qianfoshan Hospital, Shandong First Medical University, Shandong Key Laboratory of Rheumatic Disease and Translational Medicine, Shandong Institute of Nephrology, Jinan, 250014, China; Division of Endocrinology, Department of Internal Medicine, Qilu Hospital of Shandong University, Jinan, 250012, China; Department of Endocrinology and Metabology, The First Affiliated Hospital of Shandong First Medical University and Shandong Provincial Qianfoshan Hospital, Shandong First Medical University, Shandong Key Laboratory of Rheumatic Disease and Translational Medicine, Shandong Institute of Nephrology, Jinan, 250014, China; Department of Endocrinology and Metabology, The First Affiliated Hospital of Shandong First Medical University & Shandong Provincial Qianfoshan Hospital, Shandong Key Laboratory of Rheumatic Disease and Translational Medicine, Shandong Institute of Nephrology, Jinan, 250014, China

**Keywords:** Rabson-Mendenhall, INSR mutation, acanthosis nigricans, hirsutism, diagnose, meta-analysis

## Abstract

**Aims:**

Rabson-Mendenhall syndrome (RMS) is a rare autosomal, recessive disorder characterized by severe insulin resistance due to mutations in the insulin receptor (INSR) gene. This study aims to analyze the clinical features and gene mutations in RMS, which have not been extensively studied.

**Methods:**

PubMed, Embase, the China National Knowledge Infrastructure, and Wanfang were searched for “Rabson-Mendenhall syndrome” or “Black acanthosis hirsutism insulin resistance syndrome.”

**Results:**

A total of 42 cases from 33 articles were included. The body mass index ranged from 18.50 to 20.00 kg/m^2^ with an average of 16.00 kg/m^2^. There were no overweight (25.00∼29.90 kg/m^2^) or obese (≥30.00 kg/m^2^) patients. Acanthosis was present in 29 cases (29/42, 69.05%); growth retardation in 25 cases (25/42, 59.52%); dental anomalies including absence of teeth, crowding, and malocclusion in 23 cases (23/42, 54.76%); and hirsutism in 17 cases (17/42, 40.48%). The average glycosylated hemoglobin was 9.35%, and the average fasting blood-glucose was 8.44 mmol/L; the mean fasting insulin was 349.96 μIU/mL, and the average fasting C-peptide was 6.00 ng/mL. Diabetes was reported in 25 cases (25/33, 75.76%) all of which were diagnosed before 23 years old. All 42 patients had recorded gene mutations, with 22 patients (22/42, 52.38%) having ≥ 2 mutations and 20 cases (20/42, 47.62%) having only 1 mutation. No statistical differences were found in clinical features and laboratory parameters between patients with different mutations.

**Conclusion:**

The study indicates that RMS should be considered in young patients with hyperinsulinemia, hyperglycemia with low weight, acanthosis nigricans, growth retardation, dental anomalies, and hirsutism.

The human insulin receptor is composed of 2 extracellular α subunits and 2 transmembrane intracellular β subunits. The α and β subunits of the insulin receptor are encoded by a single gene (INSR) located at p13.3→p13.2 on chromosome 19's short arm. This gene consists of 22 exons and 21 introns [1]. Mutations in the INSR gene have been associated with various inherited insulin resistance syndromes, such as leprechaunism, Rabson-Mendenhall syndrome (RMS), and type A insulin resistance [2]. In 1956, Rabson and Mendenhall initially described a rare autosomal recessive inherited disease in 3 siblings [3], characterized by severe insulin resistance and fluctuations in blood glucose.

The clinical manifestations of Russell-Silver syndrome (RMS) encompass various physical characteristics such as coarse facial features, acanthosis nigricans, hirsutism, dental abnormalities, and growth retardation [4, 5]. The rarity of RMS often leads to misdiagnosis, and a comprehensive review of this condition is currently lacking. Consequently, this study aims to provide a summary of the clinical features and genetic mutations associated with RMS, with the intention of facilitating early diagnosis by healthcare practitioners.

## Subjects and Methods

### Data Sources and Study Patients

**Figure 1.**
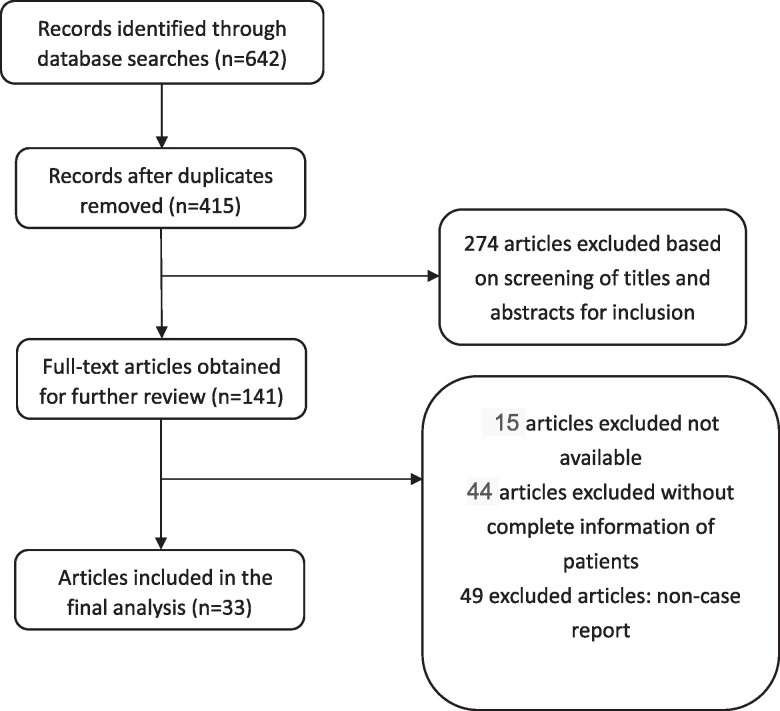
Literature review inclusion process. The number of records identified through database searches was 642. After removing duplicates and unrelated research there were 141 references left. After screening according to inclusion criteria, a total of 33 articles were ultimately included in the study.

PubMed, Embase, the China National Knowledge Infrastructure, and Wanfang databases were systematically searched from their inception until August 7, 2023, without any language limitations. The search strategies employed the following terms: “Rabson-Mendenhall syndrome” or “Black acanthosis hirsutism insulin resistance syndrome.” All included studies fulfilled the following eligibility criteria: (1) patients were definitively diagnosed with RMS through DNA testing and clinical assessment; (2) the references provided either comprehensive clinical data of the probands or documented mutations in the INSR gene. The flow chart showed the identification articles included and the reasons for their exclusions (Fig. 1).

The present study examined various clinical and laboratory variables, including (1) country and area of origin; (2) age at the time of diagnosis; (3) sex; (4) body mass index (BMI); (5) familial history; (6) clinical characteristics; (7) diabetes therapy, encompassing oral hypoglycemic agents, insulin, and dietary interventions; (8) laboratory test results at the time of diagnosis, encompassing fasting plasma glucose, fasting insulin, 2-hour postprandial plasma glucose, 2-hour postprandial insulin, glycosylated hemoglobin; and (9) identification of amino acid substitutions and mutations, along with their respective types and locations within each gene.

### Statistical Analyses

The demography, clinical, and blood indicators of patients were described utilizing simple summary statistics. Variables were analyzed by *t*-test and Wilcoxon signed rank test. Spearman's correlation analysis was used for bivariate analysis. All tests were 2-sided, and a *P*-value <.05 was considered statistically significant. Statistical analysis was performed using the Statistical Package for the Social Sciences version 26 for Windows.

### Drawing Tools

All the tables and figures were produced by Excel and GraphPad Prism 8.

## Results

### Clinical Characteristics of RMS

**Figure 2.**
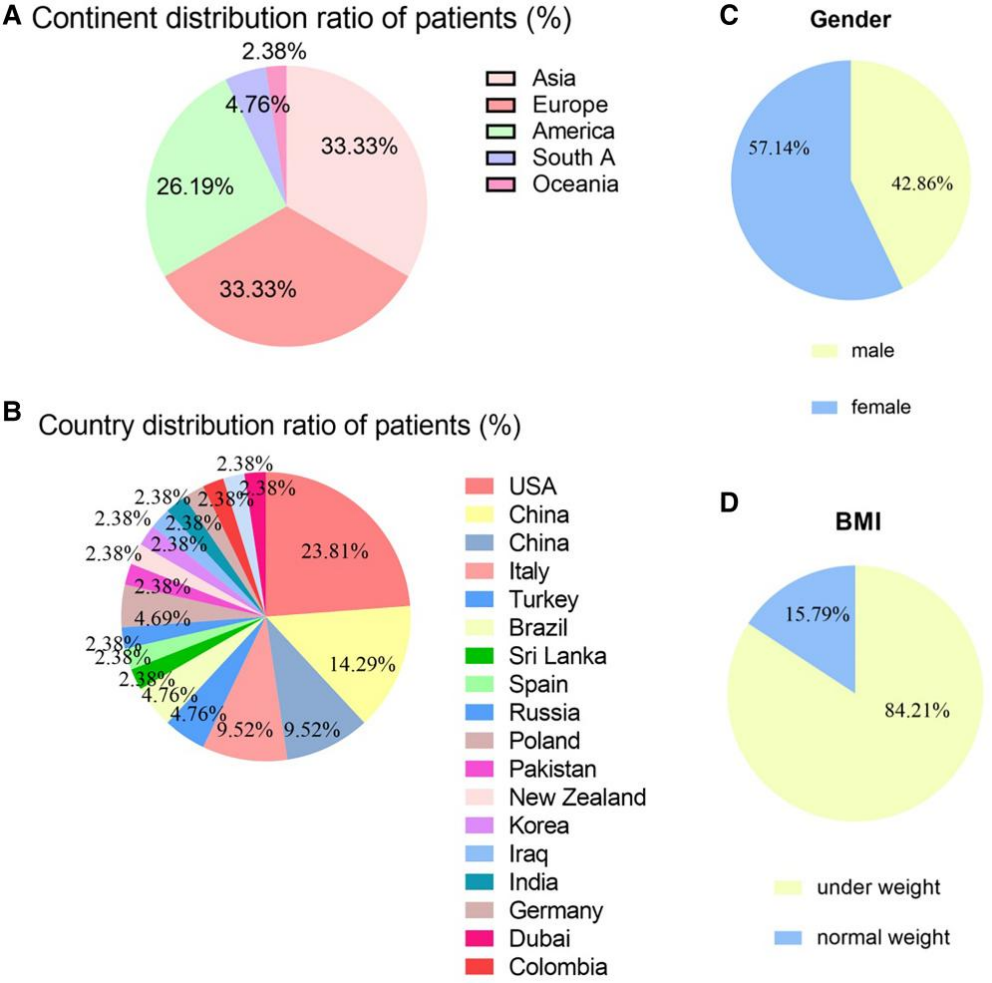
Geographical, national, sex, and body mass index distribution. (A) The patient is located in Asia (33.33%), Europe (33.33%), North America (26.19%), South America (4.76%), and Oceania (2.38%). (B) In terms of specific countries, the United States, China, the United Kingdom, Italy, Turkey, and Brazil accounted for 23.81%, 14.29%, 9.52%, 9.52%, 4.76%, and 4.76% of cases, respectively. (C) Twenty-four patients were females (24/42, 57.14%) and 18 (18/42, 42.86%) were males. (D) Sixteen patients (84.21%) were underweight (<18.50 kg/m^2^) and 3 (15.79%) were normal weight (18.50 ∼25.00 kg/m^2^), while no patient was overweight (25.00∼29.90 kg/m^2^) or obese (≥30.00 kg/m^2^).

A total of 33 studies were included in the analysis, encompassing a sample of 42 individuals diagnosed with RMS. The largest proportion of cases was found among Asians (14/42, 33.33%) and Europeans (14/42, 33.33%), followed by North Americans (11/42, 26.19%), South Americans (2/42, 4.76%), and individuals from Oceania (1/42, 2.38%). In terms of specific countries, the United States, China, the United Kingdom, Italy, Turkey, and Brazil accounted for 23.81%, 14.29%, 9.52%, 9.52%, 4.76%, and 4.76% of cases, respectively (Fig. 2A and 2B).

Twenty-four patients were females (24/42, 57.14%) and 18 (18/42, 42.86%) were males (Fig. 2C). BMI was available in 19 patients. Their BMI ranged from 8.50 to 20.00 kg/m^2^ with an average of 16.00 kg/m^2^; among them, 16 patients (16/19, 84.21%) were underweight (<18.50 kg/m^2^) and 3 (3/19, 15.79%) were normal weight (18.50 ∼25.00 kg/m^2^), while no patient was overweight (25.00∼29.90 kg/m^2^) or obese (≥30.00 kg/m^2^), according to the World Health Organization standard (Fig. 2D).

Thirteen patients had family histories accessible: 46.15% were consanguineous; 53.85% were not; and 7 (53.85%) had a history of diabetes, hirsutism, or acanthosis nigricans in their families. The age range for the 36 patients for whom data were available was 0.00 to 23.00, with an average age of 9.41 (Supplementary Table S1) [6]. Of the 8 patients for whom data were available, 37.50% (3/8) had a history of preterm birth, whereas 62.50% (5/8) were born within the expected time.

Acanthosis nigricans (29/42, 69.05%), growth retardation (25/42, 59.52%), dental anomalies (edentulous, dental crowding, and malocclusion) (23/42, 54.76%), hirsutism (17/42, 40.48%), large genitalia (8/42, 19.05%), hypertrophy of nails (6/42, 14.29%), and a protuberant abdomen (5/42, 11.90%) are some of the distinct physical signs of probands that were discovered in our study (Supplementary Table S2) [7]. Twelve patients (12/14, 85.71%) had kidney disease, with nephrocalcinosis (8/12, 66.67%), proteinuria (2/12, 16.67%), and hydronephrosis (1/12, 8.33%) being the most common conditions. In addition, 4 patients had hypoglycemia reported, and 26 patients (26/36, 72.20%) had diabetes. The aforementioned symptoms occurred at a median age of 2 years but can be seen late in young adulthood.

A mean glycosylated hemoglobin of 9.35% was measured in 27 patients (normal range: 4.00-6.00%). The median fasting blood glucose was 8.44 mmol/L (normal range: 3.90-6.10 mmol/L), including data from 28 participants. Twenty-six individuals had access to fasting insulin; all had high levels, ranging from 114 to 861 uIU/mL with a median of 300.00 (normal range: 5.00-20.00 uIU/mL). Eighteen patients had fasting C-peptide measurements, with a median of 6.00 ng/mL (normal range: 1.10-4.40 ng/mL). Furthermore, 12 patients had their 2-hour postprandial blood glucose measured, with a median value of 11.15 mmol/L (normal range: 4.40-7.80 mmol/L). The bases indicated here are expressed in Supplementary Table S1 [6].

Twenty-six probands received the following treatments: 12 patients (15/26, 57.69%) received oral hypoglycemic medicines; 10 patients (9/26, 34.62%) received both insulin and oral hypoglycemic medicines. Two patients (2/26, 7.69%) received insulin monotherapy. With the exception of a 14-year-old child who passed away from pulmonary hypertension, all patients were still alive.

### Gene Mutations in RMS

**Figure 3.**
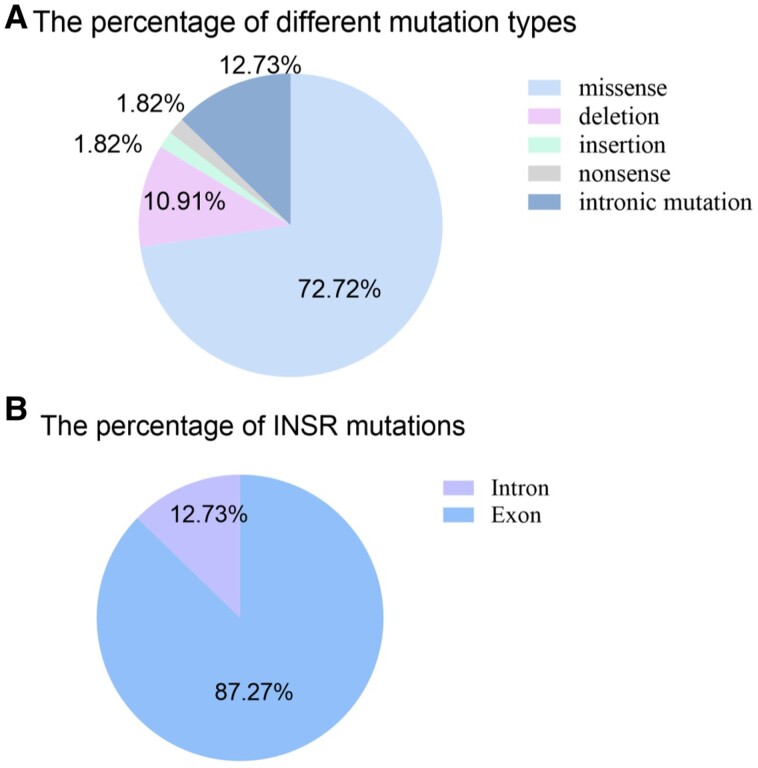
(A) The percentage of different mutation types: a total of 55 distinct INSR gene mutations, including missense (72.72%), deletion (10.91%), insertion (1.82%), and nonsense (1.82%) variants. (B) The percentage of locations on chromosome mutations: mutations were located in (87.27%) and out (12.73%) of the coding area.

Twenty of the 42 patients (20/20/42, 47.62%) had only 1 gene mutation, while 22 patients (22/42, 52.38%) had records of ≥ 2 mutations. There were no statistically significant differences between the laboratory indicators of patients with heterozygous mutations and those with a single mutation in our analysis (Supplementary Table S3) [8]. A total of 55 distinct INSR gene mutations, including missense (40/55, 72.72%), deletion (6/55, 10.91%), insertion (1/55, 1.82%), and nonsense (1/55, 1.82%) variants, were found in (48/55, 87.27%) and out (7/55, 12.73%) of the coding area (Fig. 3). With 7.27 (4/55)% of the cases, the p.Glu238Lys mutation was the most prevalent mutation. There were 34 exons found in the chromosome, with exon 2 appearing 7 times and being the most common exon overall. Laboratory indicators were also analyzed in patients with different mutant exons; no statistics difference was found (Supplementary Table S4) [9]. Gene mutations are recorded in Supplementary Table S5 [10].

## Discussion

Since RMS was first described, there have been some reports about it [11]. However, there is currently no systematic analysis of this disease. Our study summarized the clinical characteristics and genetic mutations of 42 patients. It indicates that patients with RMS have the following clinical characteristics: (1) hyperinsulinemia (100.00%); (2) nonobesity (100.00%) or underweight (84.21%); (3) high incidence of acanthosis nigricans (69.05%), growth retardation (59.52%), dental anomalies (54.76%), and hirsutism (40.48%).

RMS is a disease caused by mutations of INSR. The INSR gene is located in 19p13.2-13.3, with a full length of 170 kb, composed of 22 exons and 21 introns. It is a tetramer of 2α and 2β subunits. Exons 1 to 11 encode α subunits, and exons 12 to 22 encode β subunits [1]. Kadowaki et al [12] indicated that INSR mutations retarded the posttranslational processing of the receptor and transportation of the receptor to the plasma membrane, thereby reducing the number of receptors [13] on the cell surface. The mutation also caused a reduction in the affinity of the receptor to insulin [12] so that the mutation of INSR led to the insulin resistance of the target cell.

Insulin resistance is defined as the inability of target tissues to mount a normal coordinated glucose-lowering response at a normal plasma insulin level, including suppression of endogenous glucose production, inhibition of lipolysis, cellular uptake of available plasma glucose, and net glycogen synthesis. It is considered to be a driving factor in many diseases such as metabolic syndrome, nonalcoholic fatty liver disease, atherosclerosis, and type 2 diabetes [14, 15]. To overcome the suboptimal action in insulin-targeting tissues, organisms have to produce a large amount of insulin; thereby hyperinsulinemia develops. Hyperinsulinemia is typically observed early in the lives of RMS patients [16, 17]. A previous study reported an RMS patient who initially had hyperinsulinemia, but his insulin levels gradually decreased, and finally the proband died due to ketoacidosis [1].

Previous studies have found that high insulin levels contribute to body fat accumulation, leading to an increase in BMI [18]. However, our study found that all RMS patients had hyperinsulinemia but were not obese, and some were even underweight. On the one hand, INSR encodes 2 isoforms (INSR-A and INSR-B) depending on the exclusion or inclusion of 12 amino acids in the C-terminal domain, respectively. INSR-A is predominantly expressed in undifferentiated cells and contributes to prenatal development and tissue growth, whereas INSR-B is enhanced in postmitotic and differentiated cells, largely responsible for the systemic metabolic effects of insulin in adults [19]. The role of the different INSR isoforms in the development and function of human adipocytes has not yet been fully clarified. However, the distinct distribution of INSR isoforms on the cell surface may lead to different metabolic effects of insulin in adipocytes and other cells [20]. Insulin could increase the uptake of glucose by recruiting glucose transporters to the plasma membrane of adipose cells and potently stimulate lipogenesis by activating lipogenic and glycolytic enzymes through covalent modification [21]. At the same time, insulin can also inhibit the activity of lipase and reduces fat breakdown [22]. On the other hand, insulin resistance can lead to hyperinsulinemia. However, the reduced number of insulin receptors and decreased affinity with insulin detract from the weight gain effect of insulin, which may result in hyperinsulinemia and nonobesity in RMS patients [23].

Our study showed that all patients were diagnosed as RMS patients before 23 years old with an average of 9.41 years old. Longo et al reported a minimum survival time of 9 ± 1.4 years for RMS patients, which is much lower than that for type 2 diabetes patients [24]. It was a pity that our study did not analyze the survival times of patients due to the lack of available information.

Acanthosis nigricans results from long-term exposure of keratinocytes to insulin [25]. High levels of insulin stimulate IGF receptors, thereby promoting the proliferation of keratinocytes. As mentioned, the short isoform (INSR-A) is expressed in undifferentiated cells and contributes to prenatal development and tissue growth.

It was found that RMS patients were likely to grow slowly. Height and weight below the median of the normal reference values for children of the same age and sex, minus 2 SDs or below the third percentile, are considered as growth retardation [26]. The relationship of growth retardation and INSR mutations is still unknown. However, high insulin levels in patients may interact with IGF-I receptors and provide a feedback mechanism for reducing GH secretion [27].

The exact mechanisms responsible for dental problems of RMS patients remain unclear. However, it may be relative to hyperglycemia and hyperinsulinemia environments. A study showed that disturbances in the developing occlusion may be caused by delayed tooth eruption, accelerated tooth eruption, or altered sequence of eruption [28]. Proinflammatory caused by diabetes contributed to a reduction in both the quality and quantity of peri-tooth bone and shortens the eruption distance [28]. A previous study has reported that children with diabetes mellitus exhibit accelerated tooth eruption corresponding to a late stage of tooth eruption [29]. In addition, optimal salivary flow rate contributes to a protective environment against dental caries and periodontitis. However, hyperglycemia increased the risk of hyposalivation and thereby was related to caries and periodontitis [30]. When it comes to children in a period of growth, the development of teeth would be influenced. A previous study found that after insulin treatment, the proliferation ability of osteoblasts was weakened and the number of osteoblasts was significantly reduced [30]. However, the exact effect of excessive insulin on tooth development remains unknown and requires further research.

Some case reports mention treatment methods for RMS patients, such as oral metformin, SGLT2, insulin injection, concentrated insulin, concentrated (U-500) insulin, metreleptin (recombinant human methionyl leptin), and recombinant IGF-1. However, due to the rarity of the disease, there are currently no randomized controlled trials for related treatments, making it difficult to accurately evaluate the effectiveness of various treatment methods. Conventional diabetes treatments such as lifestyle modification, insulin or secretagogues, and insulin sensitizers are insufficient for achieving adequate glycemic control in most RMS patients, and their extreme insulin resistance limits the efficacy of high-dose insulin [31, 32]. Novel agents have been attempted. Metreleptin improved glycemia over 1 year in RMS patients [33]. Leptin is a major signal of satiety, and metreleptin is a kind of recombinant methionyl human leptin, which has been shown to improve insulin-stimulated hepatic and peripheral glucose metabolism in severely insulin-resistant lipodystrophic patients. Metreleptin subcutaneously injection in patients with congenital leptin deficiency and lipodystrophy decreased food intake and energy storage and altered central nervous system activity in regions associated with hunger and satiety, which might be the mechanisms by which metreleptin improves glucose in RMS [33, 34]. Besides, in rodent models of lipodystrophic syndromes, several mechanisms may be relevant. This includes activation of 5-AMP kinase, which will increase insulin sensitivity. In addition, leptin therapy may affect insulin receptor substrates, such as insulin-2, directly or indirectly by reducing endogenous hyperinsulinemia. However, there are no clear rodent models for RMS, which make it difficult to prove the efficiency of the therapies [35, 36].

Our study has several limitations. First, all articles were limited to the literature with available diabetes-related indicators, which might lead to selection bias. Second, due to the low prevalence of RMS, it is challenging to analyze some rare clinical manifestations. Therefore it is possible to get a negative result indicating that single or multiple mutations were not significant in indicating the severity of the disease. Further research is needed to better understand RMS.

In summary, RMS is often misdiagnosed due to its low prevalence, and there is currently no comprehensive review available. Our study systematically analyzed the clinical features and genetic mutations of 42 patients in 33 articles. For the young with hyperinsulinemia, hyperglycemia with low weight, acanthosis nigricans, growth retardation, and dental anomalies, RMS should be considered, and genetic testing is suggested in order to differentiate RMS from other types of insulin resistance syndromes.

## Disclosures

The authors declare that the research was conducted in the absence of any commercial or financial relationships that could be construed as a potential conflict of interest.

## Data Availability

The original contributions presented in the study are included in the article/Supplementary Material. Further inquiries can be directed to the corresponding author.
